# A Trial-Based Cost-Effectiveness Analysis of Erlotinib Alone versus Platinum-Based Doublet Chemotherapy as First-Line Therapy for Eastern Asian Nonsquamous Non–Small-Cell Lung Cancer

**DOI:** 10.1371/journal.pone.0055917

**Published:** 2013-03-08

**Authors:** Siying Wang, Liubao Peng, Jianhe Li, Xiaohui Zeng, Lihui Ouyang, Chongqing Tan, Qiong Lu

**Affiliations:** 1 Department of Pharmacy, the Second Xiangya Hospital of Central South University, Changsha Hunan, China; 2 School of Pharmaceutical Sciences, Central South University, Changsha Hunan, China; University of Texas Southwestern Medical Center at Dallas, United States of America

## Abstract

**Introduction:**

Lung cancer, the most prevalent malignant cancer in the world, remains a serious threat to public health. Recently, a large number of studies have shown that an epidermoid growth factor receptor-tyrosine kinase inhibitor (EGFR TKI), Erlotinib, has significantly better efficacy and is better tolerated in advanced non-small cell lung cancer (NSCLC) patients with a positive EGFR gene mutation. However, access to this drug is severely limited in China due to its high acquisition cost. Therefore, we decided to conduct a study to compare cost-effectiveness between erlotinib monotherapy and carboplatin-gemcitabine (CG) combination therapy in patients with advanced EGFR mutation-positive NSCLC.

**Methods:**

A Markov model was developed from the perspective of the Chinese health care system to evaluate the cost-effectiveness of the two treatment strategies; this model was based on data from the OPTIMAL trial, which was undertaken at 22 centres in China. The 10-year quality-adjusted life years (QALYs), direct costs, and incremental cost-effectiveness ratio (ICER) were estimated. To allow for uncertainties within the parameters and to estimate the model robustness, one-way sensitivity analysis and probabilistic sensitivity analysis were performed.

**Results:**

The median progression-free survival (PFS) obtained from Markov model was 13.2 months (13.1 months was reported in the trial) in the erlotinib group while and 4.64 months (4.6 months was reported in the trial) in the CG group. The QALYs were 1.4 years in the erlotinib group and 1.96 years in the CG group, indicating difference of 0.56 years. The ICER was most sensitive to the health utility of DP ranged from $58,584.57 to $336,404.2. At a threshold of $96,884, erlotinib had a 50%probability of being cost-effective.

**Conclusions:**

Erlotinib monotherapy is more cost-effective compared with platinum-based doublets chemotherapy as a first-line therapy for advanced EGFR mutation- positive NSCLC patients from within the Chinese health care system.

## Introduction

Lung cancer, the most prevalent malignant cancer in the world, was responsible for 13% (1.6 million) of the total cancer cases and 18% (1.4 million) of the deaths in 2008 [1]. It is the No. 1 killer among male cancer patients and the No. 2 killer among female cancer patients. The most common lung cancer is non-small cell lung cancer (NSCLC), which accounts for approximately 87% of all the diagnosed lung cancer cases.

Currently, platinum-based doublet chemotherapy–combinations of the third-generation cytotoxic drugs (gemcitabine, paclitaxel, docetaxel, pemetrexed, and vinorelbine) and platinum is generally used as the first-line therapy [2].However, none of specific platinum-based doublets mentioned above has better efficacy than the others [3]–[6]. Advances in targeted therapy have provided us with new treatment options for this disease. However, chemotherapy combined with an EGFR kinase inhibitor shows no survival benefit compared with chemotherapy alone [7]–[10].Recent data suggest that patients with activating mutations in EGFR (e.g. exon 19 deletions or exon 21L858R point mutations) achieve a significantly increased benefit from EGFR TKI therapy compared with patients who lack such mutations [11]–[16]. EGFR mutations occur more frequently in Asian patients than in white patients [17]–[19].

Erlotinib(Tarceva), is an orally administered targeted agent thatwas approved for second-line therapy by American FDA in 2005. Two phase II clinical trials suggest that erlotinib is active and well tolerated as first-line monotherapy for NSCLC [20]–[21]. In addition, two multicentre, open-label randomised phase III trials demonstrated that erlotinib delivered more significant PFS benefit and was better tolerated than standard chemotherapy in patients with advanced EGFR mutation-positive NSCLC [22]–[23]. Rafael Rosell et al reported that the erlotinib group showed a significantly longer median progression-free survival (PFS) compared with a standard chemotherapy group in European patients with EGFR mutation-positive NSCLC (9.7 months vs. 5.2 months). Caicun Zhou et al reported that for Eastern Asian patients, the median PFS was 13.1 months in the erlotinib group and 4.6 months in the chemotherapy group, indicating that Eastern Asians responded more favourably to the treatment than did white patients. These findings suggest that erlotinib is important as first-line treatment for patients with advanced EGFR mutation-positive NSCLC.

There are a number of economic analyses that have examined chemotherapy as the first-line treatment for NSCLC. However, little economic evaluation has been carried out to compare platinum-based doublet chemotherapy to erlotinib monotherapy in patients with advanced EGFR mutation-positive NSCLC. Erlotinib indeed functions positively but its acquisition cost is prohibitively high for most people. Medical decision makers need information on the economic value of the new treatment for medical resource optimisation. Therefore, this study is aimed to evaluate the cost-effectiveness of carboplatin-gemcitabine (CG) chemotherapy compared with erlotinib monotherapy as a first-line therapy for patients with EGFR mutation-positive NSCLC.

## Materials and Methods

This study was based on the data and information from the OPTIMAL trial [23], which was undertaken at 22 centres in China. A Markov model was developed to evaluate the cost-effectiveness of the two treatment strategies.

### Decision Model Structure

**Figure 1:**
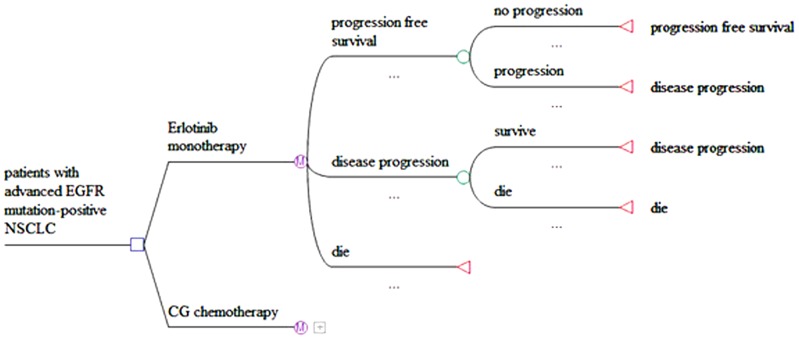
Markov model tree.

The cost-effectiveness model of advanced NSCLC involved three mutually exclusive health states: PFS, disease progression (DP) and death. Fig. 1 shows the structure of the model. At the starting point of the model, all of the patients were in a PFS and received one of treatments below as soon as they entered the PFS state:

1. 150 mg/day erlotinib until disease progression or unacceptable toxic effects and
2. Carboplatin-gemcitabine chemotherapy (carboplatin was administered intravenously under AUC = 5 on day 1, and gemcitabine was administered intravenously at 1000 mg/m^2^ on days 1 and 8) for 4 cycles.

The cycle length was 3-weeks for both groups. The transitioning probabilities of the patients between states for each cycle were estimated over a 10-year time horizon, which was chosen to reflect the nature of patients and to obtain the appropriate life expectancy in this patient population. During each 3-week cycle, the patients remained in a PFS, progressed to a DP state, or died. Once he/she entered the DP state, a patient could either remain in this state or die. The death state absorbed those patients who died from advanced NSCLC or from any other causes.

From the perspective of the Chinese health care system, we use Markov model to estimate the cost, life expectancy(LY) gained and quality-adjusted life-year (QALY) gained for both groups. The cost-effectiveness outcomes of both regimens are presented as incremental cost-effectiveness ratios (ICERs).

### Clinical data

The clinical data were derived from phase III clinical trials [23], which provided the necessary information regarding the efficacy and safety of both groups. The PFS, the primary end point of the study, was significantly longer in the erlotinib group than in the CG group (median PFS: 13.1 months vs. 4.6 months). The hazard ratio (HR) for the erlotinib group, compared with the CG group, was 0.16 (95% CI: 0.10 to 0.26; P<0.0001). We used R for Statistical Computing(R Foundation, Wien, Austria) to calculate the transition probabilities and simulate Weibull curves which then fitted to the Kaplan-Meier curves from OPTIMAL trial. Validity of the Markov model was assessed by comparing the median PFS obtained from the model to that obtained from the OPTIMAL trial.

### Costs

In this analysis, only the direct costs were estimated from the perspective of Chinese health care system. The costs used in the model consisted of the mean costs in the PFS state per cycle and mean costs in the DP state per cycle. The mean costs in the PFS state per cycle included the trial treatment costs (TT costs) and the managing adverse events costs (MAE costs). The TT costs were made of the drug costs (chemotherapy or erlotinib), the administration costs and the adjunctive care costs.

The drug costs were estimated by multiplying the unit acquisition drug costs by the number of administration cycles. The eligible patients received oral erlotinib 150 mg/day. For the chemotherapy group patients, carboplatin was administered intravenously under AUC = 5 on day 1, and gemcitabine was administered intravenously at 1000 mg/m^2^ on days 1 and 8. The erlotinib treatment continued until the disease progressed or unacceptable toxicity appeared. Chemotherapy was repeated every 3 weeks for up to 4 cycles unless the appearance of disease progression or an unacceptable level of toxicity. We assumed a 59-year-old patient with an average weight of 65 kg and a 1.72 m^2^ body surface area [24]. We assumed that none of the drug was wasted. The drug administration costs were estimated by summing all of the unit cost of drug administration per cycle. The adjunctive care costs were estimated by summing all of the unit cost of adjunctive care per cycle. All of the unit costs of TT costs are shown in table 1.

**Table 1:** Unit cost of TT costs.

Resource	Unit cost($)
**Drug costs**	
carboplatin(100 mg)	**11.71**
Gemcitabine(200 mg)	**79.06**
erlotinib(150 mg*7)	**657.05**
**Administration costs**	
Grade I nursing per day	**2.2**
Grade II nursing per day	**1.17**
Arteriovenous catheter nursing per day	**0.88**
Material per set	**10.5**
Preparation of chemotherapeutics per set	**1.5**
**Supportive care costs**	
Ondanstron(4 mg*2 ml)	**9.7**
Pantoprazole(40 mg)	**6.6**
Dexamethasone(5 mg)	**0.4**

Grade I nursing:It is intensive nursing care. Nurses visits every one hour. Nurses not only understand the disease and treatment, but also give help to patients with daily life. Nurses are required to help patients change position, take a sponge bath, cut fingers (toes) according to the disease condition.

Grade II nursing:It is not intensive nursing care. Nurses visits every two hour. Nurses are required to help patients if disease conditions of patients are change or patients have some special requirements.

Managing adverse events focused on managing neutropenia, thrombocytopenia and anaemia, all of which are considered to have a significant impact on cost. These costs have all been reported in the literature [24]–[25]. The rate of these adverse events are shown in table 2.

**Table 2:** Rate of those adverse events.

	Neutropenia	Thrombocytopenia	Anaemia
Elotinib	0	0	0
CG	42%	40%	9%

It was appropriate to remove any items that make no difference between the two regimens, because ICER depends upon incremental differences in the costs and outcomes between the two interventions. Therefore, the costs of x-rays, blood tests and laboratory tests, as well as the costs of follow-up outpatient medical review were not considered. Due to the risk of significant harm to the bone marrow, blood tests are more frequently indicated in the CG group. Therefore, the differential costs of blood tests between the two groups were considered.

In this model, the costs for patients in the CG chemotherapy group included the TT costs of CG chemotherapy and the costs of managing CG-chemotherapy associated adverse events over the course of 4 cycles. Starting with the fifth cycle, their treatment was transferred to the best support care (BSC), if the patients still remained in the PFS state. The costs of BSC were obtained from the public literature [24]. The costs for patients in the erlotinib group included the drug costs of erlotinib as erlotinib is an orally administered targeted agent that is applied for 7 cycles. Starting with the eighth cycle, the costs for patient receiving erlotinib are zero due to the donations of Roche China. The company had promised that all eligible Chinese patients (EGFR mutation-positive NSCLC) would be able to use erlotinib free of charge after being treated with erlotinib continuously 5 months. We obtained the mean costs of the DP state per month indirectly from the public literature [26]. Zeng et al reported that the mean cost of treatment for advanced NSCLC patients in the DP state was approximately US $14,519; dividing this value by 12 yielded $1,209.96, which was the mean cost of the disease progression state per month.

In the model, the costs were discounted at 3% annually to account for the current value. The costs were expressed in U.S. dollar ($) and the price year was 2010.

### Health state utilities

The health utilities for each state in this model were obtained from the public literature. These values represented the preferences of patients for various health states [27]–[28], with scores ranging from 1(a state of perfect health) to 0(death). The base health utility of PFS (with no toxicity) is 0.653. Given the main adverse events, the health utility of PFS at the CG group was adjusted to 0.56 and the health utility of PFS in the erlotinib group remained in 0.65 [29]–[31]. The utility scores for the DP state ranged from 0.673(with no toxicity) to 0.473 [30]. Furthermore, 0.47 was used for the health utility of the DP for both of the groups, as indicated in the other literature [29]–[31].

### Sensitivity analysis

Each parameter, such as transition probability, costs, and health utility, was included in the Markov model. To allow for uncertainties of those parameters and to estimate the model robustness, one-way sensitivity analysis and probabilistic sensitivity analysis were performed.

One-way sensitivity analysis assessed the impact of varying single parameters on the ICER, assuming artificial variations in one parameter at a time while holding the others constant. Its outcome was expressed as a tornado diagram. The range of each parameter is shown in table 3. We assigned the willing-to-pay (WTP) threshold at $13,527 (triple the per capita GDP of China), according to the recommendation of the WHO [32]–[34].

**Table 3:** Range of each parameter in one-way sensitivity analysis and Distribution in Probabilistic sensitivity analysis.

Parameters	Base case 2009	range		Distribution	Source
		Low	high		
**TT costs**					
cost of CG in the PFS per cycle(<5 cycle)	1599.41	1279.53	1919.29	Gamma	±20%
cost of CG in the PFS per cycle(≥5cycle)	1415.4	1022.8	2021.5	Lognormal	Bin Wu et al [24]
cost of erlotinib in the PFS per cycle(<8 cycle)	1971.1	1576.9	2365.4	Lognormal	±20%
Administration cost chemo per cyecle	54.74	43.09	64.63	Gamma	±20%
**Costs of MAE**					
Neutropenia	461.5	415.4	507.7	Lognormal	Bin Wu et al [24]
Thrombocytopenia	3395.0	3017.5	3804.6	Lognormal	Bin Wu et al [25]
Anaemia	531.7	478.5	584.9	Lognormal	Bin Wu et al [24]
**Costs of blood tests**					
CG group	10.59	8.47	12.71	lognormal	±20%
Erl group	3.53	2.82	4.24	lognormal	±20%
**cost of DP for both groups per cycle**	1209.96	967.97	1451.95	Gamma	±20%
**Risk of AE**					
Neutropenia in CG	0.42	0.34	0.50	Beta	±20%
Thrombocytopenia in CG	0.40	0.32	0.48	Beta	±20%
Anaemia in CG	0.13	0.10	0.156	Beta	±20%
**Health utilities**					
PFS of erlotinib	0.65	0.26	0.87	Beta	Carlson J et al [32]
PFS of CG	0.56	0.224	0.75	Beta	Estimated
DP	0.47	0.30	0.58	Beta	Carlson J et al [31]
**Discount rate**	0.03	0	0.08	Constant	China guideline [32]

TT costs: trial treatment costs; CG: carboplatin-gemcitabine; PFS: progression-free survival; DP: disease progression; MAE: managing adverse event.

Probabilistic sensitivity analysis provides a comprehensive assessment of the impact on model parameter uncertainty, assuming simultaneous variations in all of the model parameters on the outcome variables. It was performed using a Monte Carlo simulation with 1000 iterations and its outcomes were expressed as cost-effectiveness acceptability curve and proportion of cost-effectiveness graph. The distributions of each of the parameters are shown in table 3.

## Results

### Base case analysis

The median PFS obtained from the Markov model was 13.2 months in the erlotinib group and 4.64 months in the CG group compared with 13.1 months and 4.6 months extracted from the trial, respectively. The base-case outcomes are summarised in Table 4. The base-case outcomes are also discounted at 3% per year.

**Table 4:** Base case results discounted at 3% per year.

Results	Erlotinib	CG
**Effectiveness**		
Life expectancy(years)	2.5	4.08
PFS of life expectancy	1.27	0.43
DP of life expectancy	1.23	3.65
QALYs	1.4	1.96
PFS of QALY	0.82	0.24
DP of QALY	0.58	1.72
**Lifetime costs (US $)**		
Mean costs of managing adverse events	——	1620.951
Mean costs in PFS	14772.04	13060.35
Mean costs in DP	25335.91	75166.95
Total costs	40107.95	88227.3
**ICER(US $/QALY)**	85927.41	

QALYs: quality-adjusted life years ICER: incremental cost-effectiveness ratio Costs in 2010 US dollars.

The discounted life expectancy was 2.5 years (30 months) in the erlotinib group and 4.08 years (49 months) in the CG group, resulting in a difference of 1.58 years. The QALY was 1.4 years in the erlotinib group and 1.96 years in the CG group, resulting in a difference of 0.56 years. Those differences were due to the shorter amount of time spent in the DP state for the erlotinib group, reflecting a higher risk of death after disease progression in the erlotinib group. However, the PFS in the elotinib group was significantly longer than that in the CG group from the outcomes of the Markov model (9.84 months vs2.88 months). Therefore, we can conclude that the life quality of the patients was improved from the fact that the latter difference was less than the former (1.58years vs. 0.56 years).

The mean costs during the PFS of the erlotinib group were higher than its counterpart, due to the high acquisitive cost of elotinib and the longer amount of time remaining in PFS. However, the difference in the mean costs during PFS between the two groups was not as significant as the difference in their acquisition costs. The main contributor to this effect was the benefit that elotinib would be free of charge for patients with EGFR mutation-positive NSCLC after 5 months of erlotinib treatment. A variety of treatment regimens were used for each patient when he or she entered disease progression. Therefore, we assigned the same mean costs in disease progression state per cycle both of the groups.

Relative to CG, erlotinib resulted in $30455.28 per life year gained and $85927.41 per QALY gained.

### Sensitivity analysis

**Figure 2:**
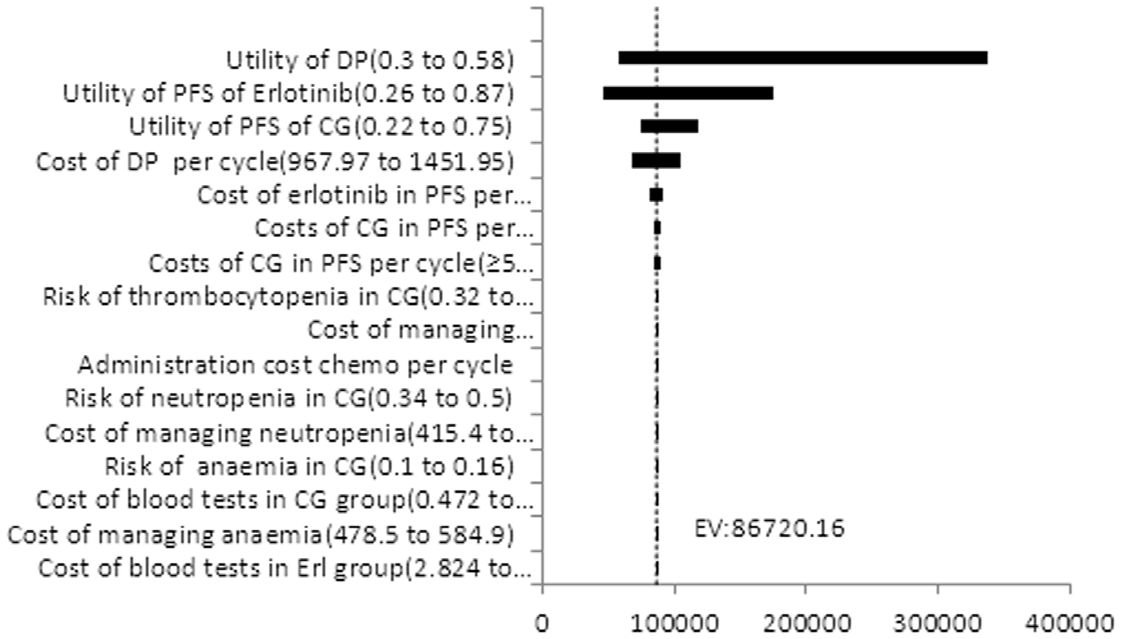
ICER Tornado Diagram. ICER: incremental cost-effectiveness ratio Costs in 2010 US dollars.

Fig. 2 shows tornado diagrams for the ICER. We can find those sensitive parameters: the health utility of DP, the health utility of PFS in the erlotinib group, the health utility of PFS in the CG group, the costs of DP per cycle, the cost of erlotinib in the PFS per cycle(<8 cycle), the cost of CG in PFS per cycle(<5 cycle) and cost of CG in the PFS per cycle(≥5cycle) in descending order. Those parameters had significant impacts on the ICER. Furthermore, the ICER was most sensitive to the health utility of DP, ranging from $58,584.57 to $336,404.2. None of the ICERs were less than the WTP threshold of $13527, nor did it change significantly when the sensitive parameters varied within the assigned ranges.

**Figure 3:**
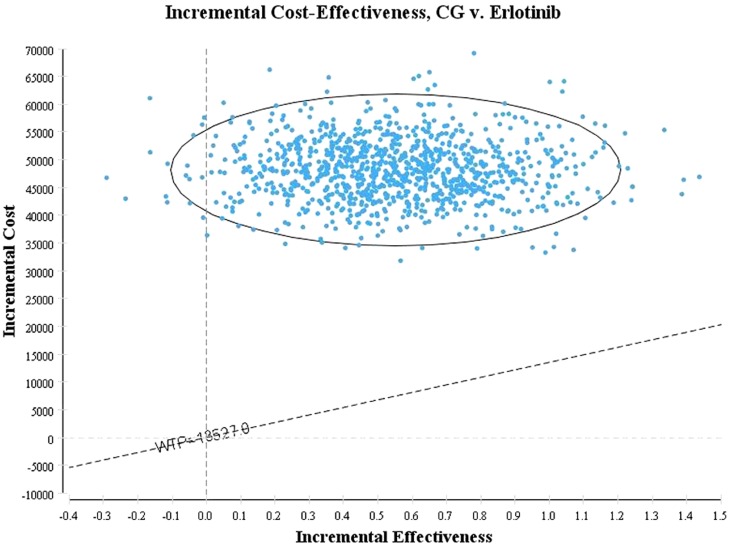
Incremental cost-effectiveness Scatter Plot.

**Figure 4:**
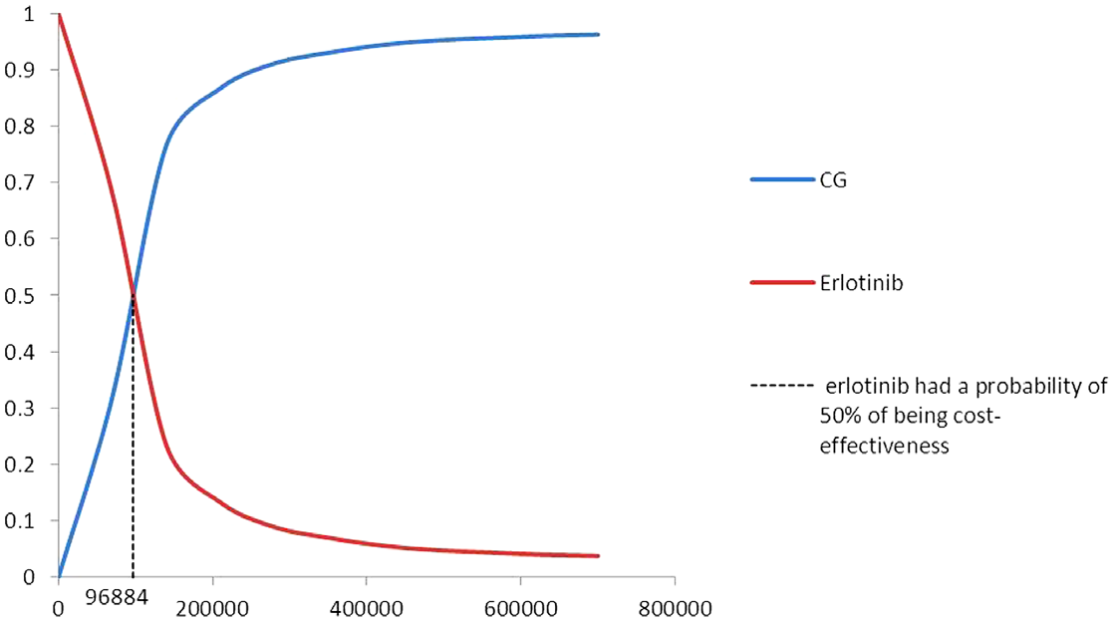
Cost-effectiveness acceptability curve of erlotinib vs.CG chemotherapy. CG chemotherapy: carboplatin-gemcitabine chemotherapy.

Fig. 3 shows the proportion of cost-effectiveness for CG compared with erlotinib alone. More than 90% of the 1000 simulation iterations fell within the northeast quadrant (CG doublets results in QALYs gains at additional costs compared with erlotinib monotherapy), whereas the other iterations were located in the northwest quadrant (CG doublets results in QALYs loss at additional costs compared with erlotinib monotherapy). The probability of meeting the WTP threshold was 0, which suggests that within the WTP threshold, 100% of the patients in the erlotinib group could achieve a better cost-effectiveness, compared with the patients in the CG group. Fig. 4 shows the acceptability curve for the erlotinib and CG strategies at various WTP thresholds in patients with advanced EGFR-positive NSCLC. At a threshold of $96,884, erlotinib exhibited a 50% probability of being cost-effective. And when the WTP threshold was≤$10,264, the erlotinib regimen was able to achieve 95% cost-effectiveness. When the WTP threshold≥$420,000, the CG regime could achieve 95% cost-effectiveness.

## Discussion

We performed a cost-effectiveness analysis of erlotinib alone compared with carboplatin-gemcitabine as first-line therapies for patients with advanced EGFR-positive NSCLC, based on the OPTIMAL trial. At the base-case, compared with the CG regimen, elotinib significantly prolonged the PFS component of life expectancy by 0.84 years (10 months), improved the PFS of the QALY by 0.58 years (7 months), despite the fact that both the whole life expectancy and the QALY of patients treated with erlotinib were less than those of counterparts. However, the quality in the erlotinib group could be improved over that in the CG group because there were no serious adverse events reported in the erlotinib group and its mode of drug delivery was more convenient. Furthermore, the costs spent in the erlotinib group were far less. There are two major contributors to this result: 1. serious adverse event management did not need to be considered and; 2.erlotinib would be free charge for eligible patients (EGFR mutation-positive NSCLC) after 5 months of erlotinib treatment because of donations made by Roche China. The ICER ($85927.41/**QALY**) indicates that erlotinib alone as the first-line therapy is cost-effective at the WTP threshold of $13527 from the perspective of the health care system in China.

Several economic studies have been performed to evaluate the use of erlotinib to treat advanced NSCLC. Erlotinib as a second- or third-line therapy for patients with NSCLC provides equivalent to slightly improved outcomes with variability in the incremental costs depending on the health system in which the analysis was performed [35]. Table 5 displays some of the economic analyses undertaken since 2008 that have estimated the cost-effectiveness of erlotinib as a second-line treatment for advanced NSCLC.

**Table 5:** Erlotinib as second-line setting economics studies in advanced NSCLC since 2008.

Country	Intervention	Comparator	ICER	Source
UK	Erlotinib	BSC	€20,711/LYG	Silke Walleser et al [36]
Germany	Erlotinib	BSC	€25,124/LYG	Silke Walleser et al [36]
Greece	Erlotinib	pemetrexed	No statistically significantly difference	Fragoulakis et al [37]
British	Erlotinib	BSC	$36,838/LYG	Cromwell et al [38]
British	Erlotinib	docetaxel	No statistically significantly difference	Cromwell et al [39]
Portugal, Italy, France, Canada, Poland	Erlotinib	docetaxel	Cost saving	Lyseng Williamson et al [40]
	Erlotinib	pemetrexed	Cost saving	
UK	Erlotinib	docetaxel	£7062.5/QALY	Lewis et al [29]
Canada	Erlotinib	BSC	$94 638/LYG	Bradbury et al [41]
Brazil	Erlotinib	docetaxel	Cost saving	Stephen et al [42]
		pemetrexed	Cost saving	
USA	Erlotinib	docetaxel	Dominant	Carlson et al [43]
		pemetrexed	dominant	

BSC: best supportive care.

This is the first study to evaluate the cost-effectiveness of an EGFR TKI alone as a first-line therapy compared with platinum-based chemotherapy. Erlotinib is a cost-effective treatment versus best supportive care when used as first-line maintenance therapy for locally advanced or metastatic NSCLC [44]. However, there are no cost-effectiveness analyses of erlotinib versus platinum-based chemotherapy as first-line therapy for EGFR mutation-positive NSCLC that have previously been published. Joan Schiller et al. reported that the average treatment-related costs of cisplatin-gemcitabine were lower than those of cisplatin-vinorelbine, cisplatin-paclitaxel and carboplatin-paclitaxel, and similar or lower than those of cisplatin-docetaxel; they also reported that none of the specific platinum-based doublets showed better efficacy than the alternative doublets. The incremental cost savings per patient of cisplatin-gemcitabine compared to cisplatin-vinorelbine ranged from €827($1094) to €2055($2718) per patient and from €1616($2138) to €5342($7066) compared to the paclitaxel-cisplatin/carboplatin from the European payer perspective [45]. Therefore, we can infer from this indirect comparison that erlotinib alone is more cost-effective than other platinum-based doublets for EGFR mutation-positive NSCLC.

This study emphasizes the importance of the EGFR mutation-positive status in identifying the appropriate patients. EGFR gene mutations are the most important predictive factors for the robust responses of NSCLC to EGFR TKI [46]–[48]. Certain clinical factors that are highly sensitive to EGFR-TKI, such as adenocarcinoma, being a non-smoker, the female gender and the Asian race [47], [49]–[50], have been reported to be associated with EGFR gene mutations Christos Chouaid et al. assessed two cost-effectiveness analyses based the GFPC 0505 study. The only difference between the two analyses was the eligible population: one included all patients and the other only examined patients who were more than 70 years old. The first study found no difference in cost-effectiveness between the two groups, whereas the later study indicated that the total costs and QALY for the erlotinib-first strategy were €27734($36686) and 0.51 year respectively, compared to €31688($41917) and 0.52 years, respectively, for the chemotherapy-first strategy [51]–[52]. Likewise, cisplatin-gemcitabine is cost-effective as a first-line therapy compared with erlotinib for advanced EGFR wild-type NSCLC [53] whereas our study showed that erlotinib is cost-effectiveness as first-line therapy compared with cisplatin-gemcitabine for advanced EGFR mutation-positive NSCLC. Therefore, distinguishing the patients with EGFR mutation-positive tumors from those with EGFR wild-type tumours is helpful to improve clinical benefit and save money on treatment costs, which is supported by several studies. The study outcomes of Josh J. Carlson et al [31], [35] suggest that EGFR pharmacogenomic testing has the potential to improve the QALY during the treatment of refractory NSCLC. Borget et al reported similar outcomes, indicating that a biologically guided strategy was slightly less expensive than the corresponding clinically guided strategy [54].

One potential strategy to improve the cost-effectiveness of erlotinib is to reduce its high acquisition cost. The unit price of erlotinib is $657.05/150 mg*7 and the treatment cost of erlotinib per cycle in PFS was $1971.1, which is too high for most Chinese people. Fortunately, due to the donations of Roche China, the treatment costs of erlotinib were considerably reduced. The company has promised that all eligible Chinese patients (EGFR mutation-positive NSCLC) would be able to use erlotinib free of charge after they have treated with erlotinib continuously for 5 months. However, erlotinib monotherapy would fail to be cost-effective in other countries that lack this special benefit.

The other way to improve the value of erlotinib is to further develop the predictive molecular biomarkers to improve the clinical effect of the EGFR TKI. Due to the existence of EGFR TKI resistance, the combination with other EGFR inhibitors would prevent EGFR TKI resistance from occurring and improve the EGFR TKI response. A recent randomized trial demonstrated that the combination of bexarotene (the first RXR-selective retinoid) with/tivantinib (a MET inhibitor) and erlotinib is effective as a treatment for *KRAS-*mutation-driven lung cancer and is well-tolerated [55]–[56].

There are three strengths of the present evaluation. Firstly, this study is the first cost-effectiveness analysis of erlotinib monotherapy compared with platinum-based chemotherapy as first-line treatment of EGFR mutation-positive NSCLC patients. Secondly, the Markov model simulated the natural progression of advanced NSCLC and closely matched the reported PFS curve and mortality. Lastly, clinical data from a head-to-head open-label phase III trial is more reliable and available to our study because this trial was undertaken at 22 centres in China and included Chinese patients.

There are several limitations of our study. Firstly, the Weibull curves simulated by R may not accurately mirror the true condition when the patients enter disease progression, due to the lack of an overall survival curve from the OPTIMAL trial. Secondly, the costs of CG in PFS per cycle(≥5cycles), which were estimated from the costs of the BSC used data from a Chinese economic evaluation of advanced gastric cancer [24]. Although these data are not head-to-head data applied to NSCLC, the costs of BSC remain similar in different cancers, according to the doctors. Lastly, all of the health utilities used in our study were extracted from foreign literature. Generally, health utilities differ among patients according to their race, religion, culture, economic background and concept of health maintenance. It is perhaps unreasonable to apply the health utilities of Western counties to Chinese people. Because of the lack of data on Chinese health utilities, we had to use data from other countries.

## Conclusion

In conclusion, this is the first study to evaluate the cost-effectiveness of an EGFR TKI in comparison with platinum-based doublets chemotherapy for advanced EGFR mutation-positive NSCLC. Erlotinib monotherapy is cost-effective compared with platinum-based doublets chemotherapy as a first-line therapy for advanced EGFR mutation- positive NSCLC patients from the perspective of the Chinese health care system. This study emphasizes the importance of the presence of an EGFR gene mutation in assessing the incremental effect and costs of erlotinib as a first-line treatment. Our study may provide a valuable reference for making informed decisions about resource allocation.
